# RFID Sensor with Integrated Energy Harvesting for Wireless Measurement of dc Magnetic Fields

**DOI:** 10.3390/s25103024

**Published:** 2025-05-10

**Authors:** Shijie Fu, Greg E. Bridges, Behzad Kordi

**Affiliations:** Department of Electrical and Computer Engineering, Price Faculty of Engineering, University of Manitoba, Winnipeg, MB R3T 5V6, Canada; fus34@myumanitoba.ca (S.F.); gregory.bridges@umanitoba.ca (G.E.B.)

**Keywords:** magnetic field sensing, RFID, RF power harvesting, HVdc transmission, HVdc system monitoring

## Abstract

High-voltage direct-current (HVdc) transmission lines are gaining more attention as an integral part of modern power system networks. Monitoring the dc current is important for metering and the development of dynamic line rating control schemes. However, this has been a challenging task, and there is a need for wireless sensing methods with high accuracy and a dynamic range. Conventional methods require direct contact with the high-voltage conductors and utilize bulky and complex equipment. In this paper, an ultra-high-frequency (UHF) radio frequency identification (RFID)-based sensor is introduced for the monitoring of the dc current of an HVdc transmission line. The sensor is composed of a passive RFID tag with a custom-designed antenna, integrated with a Hall effect magnetic field device and an RF power harvesting unit. The dc current is measured by monitoring the dc magnetic field around the conductor using the Hall effect device. The internal memory of the RFID tag is encoded with the magnetic field data. The entire RFID sensor can be wirelessly powered and interrogated using a conventional RFID reader. The advantage of this approach is that the sensor does not require batteries and does not need additional maintenance during its lifetime. This is an important feature in a high-voltage environment where any maintenance requires either an outage or special equipment. In this paper, the detailed design of the RFID sensor is presented, including the antenna design and measurements for both the RFID tag and the RF harvesting section, the microcontroller interfacing design and testing, the magnetic field sensor calibration, and the RF power harvesting section. The UHF RFID-based magnetic field sensor was fabricated and tested using a laboratory experimental setup. In the experiment, a 40 mm-diameter-aluminum conductor, typically used in 500 kV HVdc transmission lines carrying a dc current of up to 1200 A, was used to conduct dc current tests for the fabricated sensor. The sensor was placed near the conductor such that the Hall effect device was close to the surface of the conductor, and readings were acquired by the RFID reader. The sensitivity of the entire RFID sensor was 30 mV/mT, with linear behavior over a magnetic flux density range from 0 mT to 4.5 mT.

## 1. Introduction

Overhead high-voltage direct-current (HVdc) transmission lines are gaining more attention as an integral part of modern power system networks. Monitoring the magnetic field is important for non-contact current measurements and the implementation of dynamic line rating control schemes. To avoid direct electrical connection to the transmission line network, researchers and industries have utilized the magnetic field generated by the line to determine the current intensity [1,2,3]. The direct-current current transformer (DCCT) is the most accurate dc high-current measurement sensor that is commercially available [4]. However, the calibration and installation of a DCCT is challenging and complex [5,6].

Magnetic field sensors are also widely used for the monitoring of many different parameters of overhead HVdc transmission lines, such as current measurement, voltage measurement [7], sagging, galloping [8], and fault location detection [9]. Magnetic field measurement can be performed using different approaches to monitor overhead HVdc transmission lines. However, techniques that require direct contact with the HVdc conductor are challenging, and the exploration of wireless/remote sensing methods with high accuracy and a wide dynamic range is needed.

Radio frequency identification (RFID) technology is a promising and well-developed wireless communication method [10]. Many passive RFID devices have user-accessible memory, which can be used to store sensor information as well as the tag ID. As the power required by a commercial passive RFID device to respond to the RFID reader is fairly low, it can operate over a distance of several meters. When a sensor is incorporated within the tag, additional power is needed, and a rectenna, which collects RF energy from the reader, can be added to the RFID sensor [11]. An integrated RFID sensor, termed the self-power augmented RFID tag for autonomous computing and ubiquitous sensing (SPARTACUS), was presented in [12]. SPARTACUS has a 3 m communication distance and programmable memory optimized for RFID sensing. The sensor enables bidirectional communication with the reader. Many RFID-based sensors are capable of measuring the distance of the target from the reflected power level. This characteristic is common among many RFID sensors [13]. Multi-RFID sensor networks have been applied to various measurands, such as humidity [14] and food quality assessment [15]. In some RFID sensors, the measurand modulates the RFID tag’s received signal strength [16,17]. However, this approach only works well for situations that do not require a high-resolution, quantitative measurement.

RFID sensors are becoming popular in power systems due to their ability to offer wireless, contactless measurement solutions. The use of RFID reduces the need for complex wiring and maintenance in high-voltage environments. In [18,19], RFID sensors are used for the monitoring of power transformer vibration and the diagnosis of transformer conditions. The RFID sensor tag utilizes an inductive energy harvester through coupling with the transformer’s ambient AC magnetic field to power a three-axis accelerometer, which is used to wirelessly collect 3D vibration signals from different locations inside a power transformer to identify the fault location. Chipless RFID sensors have been designed for partial discharge detection. Normally, PD emits electromagnetic energy in the UHF band (300 to 3000 MHz). Researchers have investigated the possibility of capturing the radiated electromagnetic energy using antennas. When there is a PD event, the PD will induce a voltage in the sensing coil that biases a Schottky diode. This results in a change in the impedance of the RFID tag and consequently a change in the backscatter response [20,21]. In [22], a chipless RFID sensor is used to measure the time-varying electric field in a HVac asset. As the need for real-time monitoring and predictive maintenance grows, RFID-based sensing can play a key role in improving the reliability and performance of modern power systems. Table 1 provides examples of wireless sensors applied to overhead transmission line monitoring.

**Figure 1:**
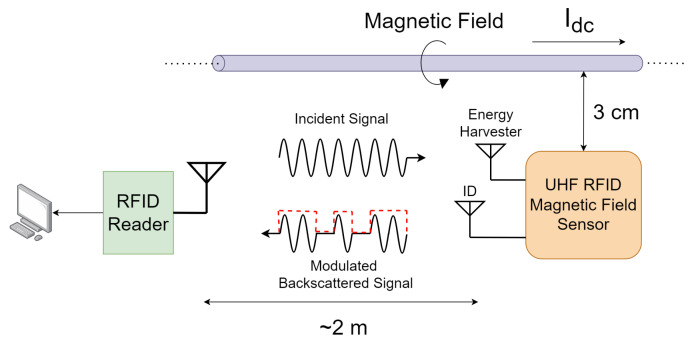
Schematic of the UHF RFID-based sensor tag for the magnetic field measurement of an overhead HVdc transmission line.

The use of a powered RFID sensor for the measurement of the magnetic field of a dc overhead transmission line was demonstrated by the present authors in a previous publication [28]. In that work, a proof of concept using RFID technology combined with a Hall effect sensor for the wireless measurement of dc magnetic field was presented. In [28], the system was not batteryless, and the Hall effect sensor and the microcontroller were continuously powered by a separate external power supply. In this paper, the design and fabrication of a batteryless RFID-based magnetic field sensor is presented. The entire RFID sensor (including the microcontroller and the Hall effect sensor) is wirelessly powered by the energy harvested from the RFID reader. The sensor ensures simplicity in operation and offers a wireless, efficient solution to measure the magnetic field on HVdc transmission line conductors. As shown in Figure 1, the sensor consists of a UHF RFID tag integrated with a Hall effect sensor, which encodes the tag’s memory with the measured magnetic field and is subsequently used to modulate the backscattered signal. The developed sensor is equipped with two antennas: one for identification and one for energy harvesting. The same reader is used to both power the RFID sensor and read the magnetic field measurement data encoded in the modulated backscattered signal. A prototype on a single PCB that contained all the components, including the RFID tag antenna and the energy harvester antenna, was fabricated and tested.

## 2. RFID System Design

**Figure 2:**
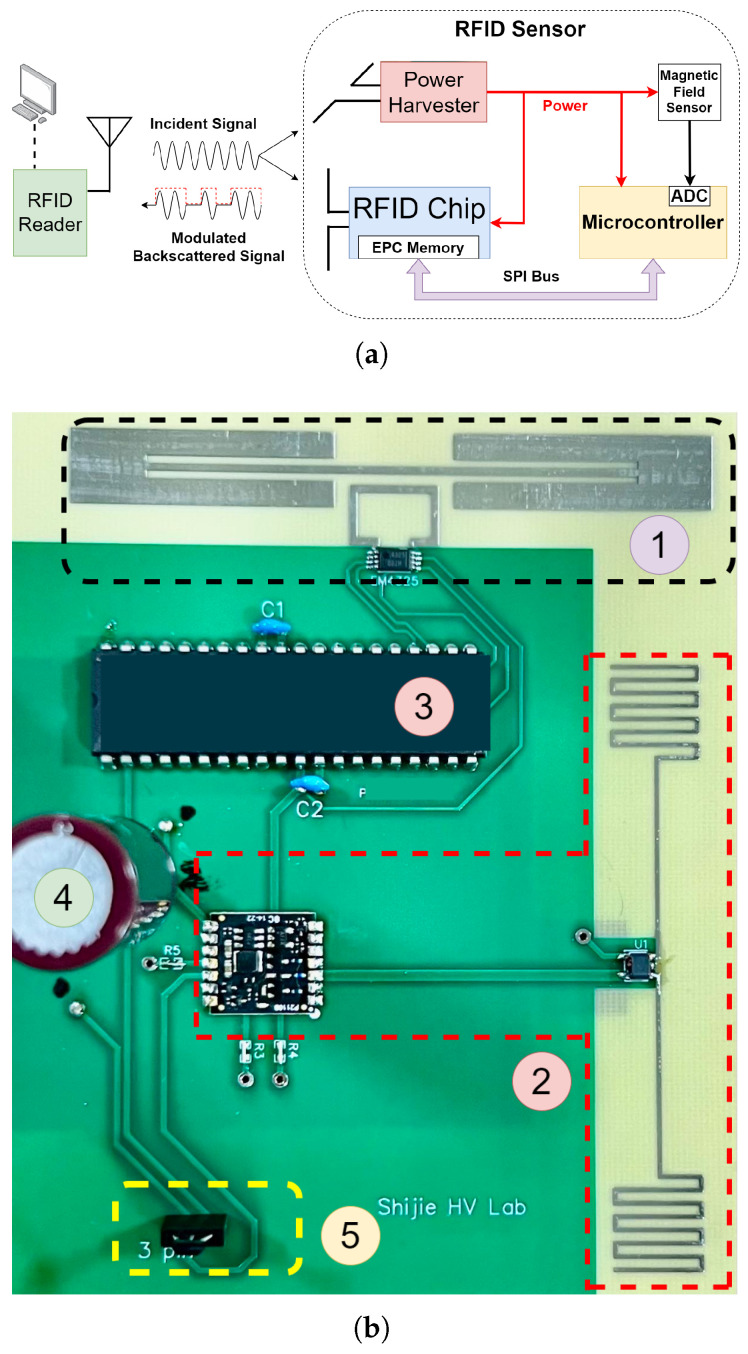
(**a**) A block diagram of the UHF RFID-based magnetic field sensor tag. (**b**) Top view of the fabricated sensor consisting of (1) passive RFID chip and its antenna, (2) power harvester and its antenna, (3) microcontroller, (4) supercapacitor, and (5) Hall effect sensor.

Figure 2a shows the block diagram of the developed sensor. It comprises a linearly polarized antenna matched to the RFID chip, a microcontroller, a magnetic field sensor, and an RF power harvester and its own antenna. The prototype of the sensor developed on a single printed circuit board (PCB) is shown in Figure 2b. This integrated configuration enables efficient communication, data processing, and magnetic field sensing within a compact, planar design. The power harvester is an integrated circuit that is used to capture energy emitted by the RFID reader. The harvested energy is converted into a stable dc output and stored in a supercapacitor. The embedded power management circuit controls the power to both the magnetic field sensor and the microcontroller and prevents excessive energy consumption. A Hall effect sensor is used to measure the dc magnetic field. When a magnetic field is present, the Hall effect sensor produces an analog voltage signal that is fed to the microcontroller’s analog-to-digital converter (ADC). The digital data reflect the strength and direction of the measured magnetic field. In this design, the microcontroller is configured to be a serial peripheral interface (SPI) master, and the RFID chip is configured as an SPI slave. (See Appendix A for details.)

The microcontroller writes the digital magnetic field information to the EPC memory of the RFID chip through the SPI. The EPC memory is a writable memory within the RFID chip where data can be stored, retrieved, and read by the RFID reader. The UHF RFID sensor can operate within a maximum distance of 2 m from the RFID reader. The Hall effect sensor in this design can measure the magnetic field flux density up to a maximum of 20 mT, which is higher than the typical dc magnetic field in the vicinity of the conductors of typical HVdc transmission lines. To measure larger magnetic fields, a different magnetic field sensor can be used.

## 3. Antenna Design and Measurement

### 3.1. UHF RFID Tag Antenna

**Figure 3:**
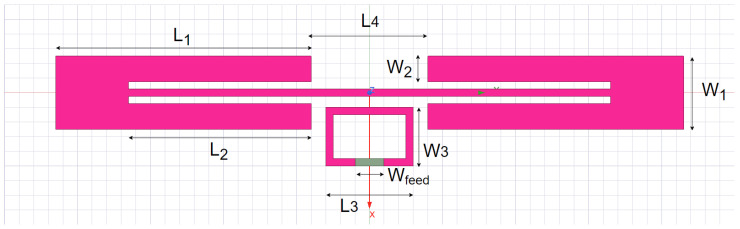
UHF RFID coplanar antenna ($L_{1}$ = 35 mm, $L_{2}$ = 25 mm, $L_{3}$ = 12 mm, $L_{4}$ = 16 mm, $W_{1}$ = 10 mm, $W_{2}$ = 3 mm, $W_{3}$ = 8 mm, $W_{\mathrm{feed}}$ = 4 mm, gap = 1.5 mm).

A key component in designing an efficient RFID sensor is its antenna. A well-matched antenna design optimizes both the read range and the communication efficiency of the sensor tag. The RFID chip used in this work, EM4325, has an input impedance of $Z_{RFIC}=7.6-j114\;\Omega$ at 915MHz [29]. A coplanar stripline (CPS) antenna [30], shown in Figure 3, is employed as a suitable choice for matching. The CPS antenna design is based on a quarter-wavelength dipole antenna, loaded by two coplanar striplines. Instead of a direct connection, a small loop is used to couple the CPS antenna to the RFID chip. The loop, in conjunction with the CPS antenna, enables the adjustment of the imaginary part of the antenna impedance while reducing the resistive part.

**Figure 4:**
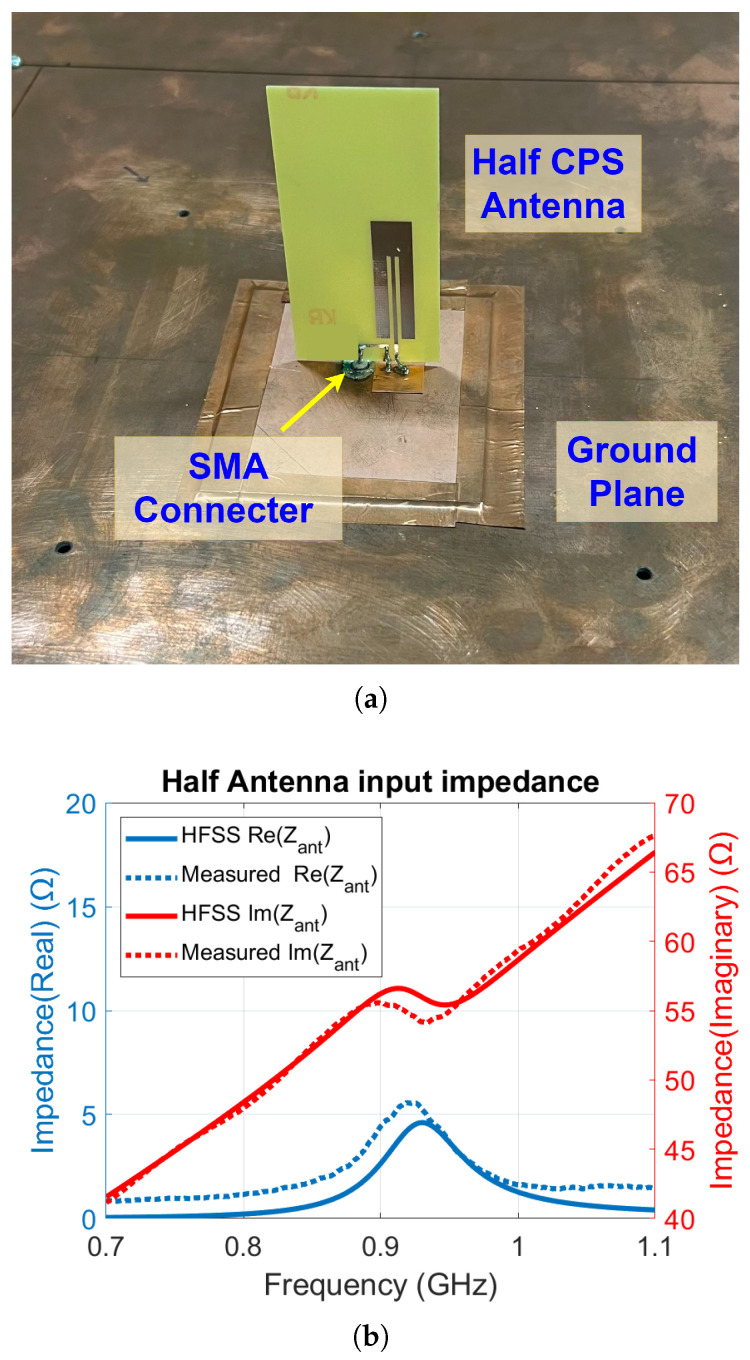
(**a**) Half-antenna test setup. (**b**) Measured and simulated input antenna impedance of the CPS half-antenna for the RFID tag. The resonance frequency of the CPS antenna is 915 MHz.

Using ANSYS HFSS, the RFID antenna was designed and simulated to have an input impedance of $Z_{\mathrm{RF}-\mathrm{Simulated}}=7.8+j113\;\Omega$ at a frequency of 915 MHz. The antenna was designed for an FR4 substrate with a thickness of 0.6 mm, relative permittivity of 4.4, and loss tangent 0.02. Once fabricated, the antenna’s input impedance was measured. As the CPS antenna is a balanced antenna, the half-antenna measurement method was used to measure its input impedance. Figure 4a shows the measurement setup, where a half CPS antenna is positioned above a ground plane that is much larger than the size of the antenna. The feeding port of the half-antenna is soldered to an SMA connector and connected to a vector network analyzer (VNA) to measure its input impedance. The measured real and imaginary parts of the CPS antenna input impedance over the frequency range 700 MHz to 1.1 GHz are shown in Figure 4b. The impedance shown corresponds to the half-antenna, which is half of the full antenna impedance. At the operating frequency of 915 MHz, the measured input impedance of the full antenna is $Z_{\mathrm{RF}-\mathrm{Measured}}=10.8+j110\;\Omega$.

### 3.2. Power Harvester Antenna

**Figure 5:**
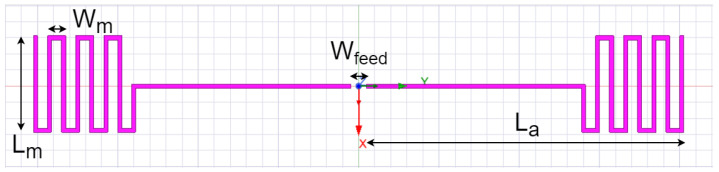
Harvester meander line dipole antenna ($L_{a}$ = 39.5 mm, $L_{m}$ = 12 mm, $W_{m}$ = 2.25 mm, $W_{\mathrm{feed}}$ = 2 mm).

The power harvester of the RFID sensor uses a meander line dipole antenna (see Figure 5) to collect RF energy emitted by the RFID reader. To avoid interference, it is placed orthogonally to the RFID antenna. The power harvester circuit, P2110B, has a 50Ω input impedance. To reduce the size of the antenna, a meander line section is added to the ends of a dipole antenna. The antenna is designed to have an input impedance with an imaginary part close to zero and a real part lower than 50Ω.

**Figure 6:**
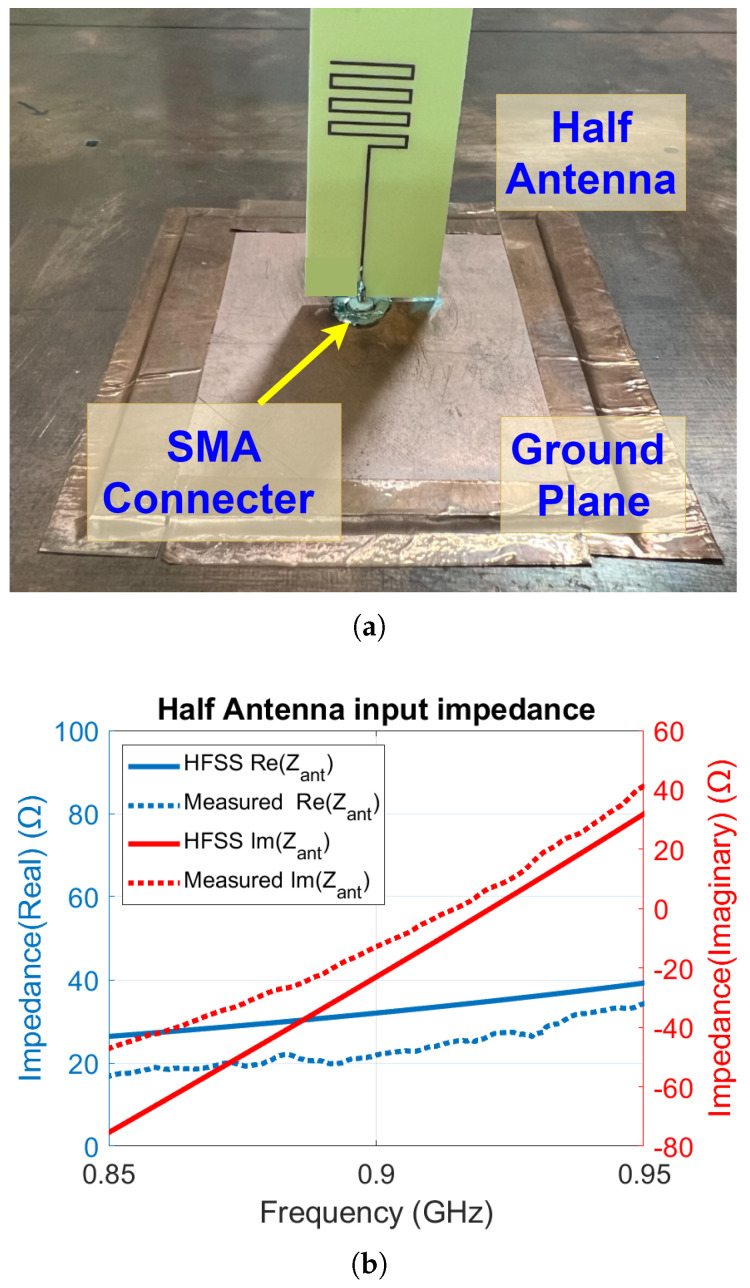
(**a**) Half-antenna test setup. (**b**) Measured and simulated input impedance of the meander line half-dipole antenna for the power harvester.

The simulated input impedance of the harvester antenna calculated using ANSYS HFSS is $Z_{\mathrm{EH}-\mathrm{Simulated}}=34.9+j0.1\;\Omega$ at a frequency of 915 MHz. The harvester antenna was fabricated on the same FR4 board as the RFID antenna. Since the power harvester antenna is also a balanced antenna, the same half-antenna method was used to measure its input impedance, as shown in Figure 6a. The measured real and imaginary parts of the half-power harvester antenna over the frequency range of 850 MHz to 950 MHz are shown in Figure 6b. At the operating frequency of 915 MHz, the measured input impedance of the full-power harvester antenna is $Z_{\mathrm{EH}-\mathrm{Measured}}=26.7+j7.0\;\Omega$. To match the balanced 26.7 Ω input impedance of the harvester antenna to the 50Ω input impedance of the power harvester, a 1:1 balun (ETC1-1-13TR) with an insertion loss of around 2dB and a quarter-wavelength transmission line are employed, as shown in Figure 2a. The length and and characteristic impedance of the line are designed to be 47 mm and 36Ω, respectively, at 915 MHz.

**Figure 7:**
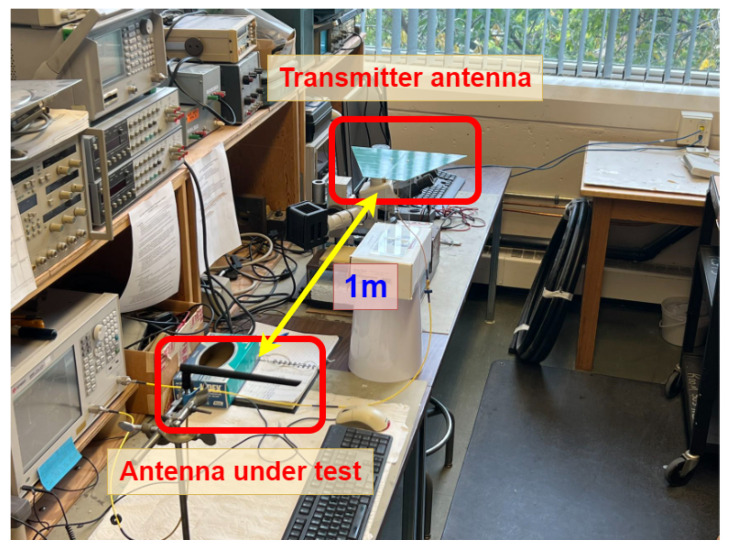
Experimental setup for antenna gain measurement.

An important parameter for the power harvester antenna is the antenna gain. For a meander line dipole antenna, the gain will be lower than that of a half-wavelength dipole antenna. To determine the gain of the harvester antenna, relative antenna gain measurement was performed. The setup is shown in Figure 7, using a 2.5 dB Yagi antenna as the transmitter and a commercial monopole antenna with an antenna gain of 0.5 dBi as a reference antenna. The gain of the harvester antenna is determined by replacing the reference antenna with a meander line antenna and measuring the difference in the transmission parameter using a VNA. The designed meander line antenna has a gain of ≈1 dBi at the operating frequency of 915 MHz.

## 4. Magnetic Field Measurement and Data Transmission

**Figure 8:**
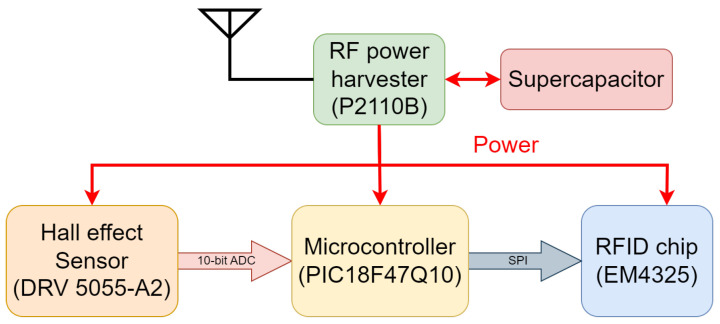
Functional schematic of the relationship between the RFID tag components, showing the power distribution and data communication path.

In this section, the interfacing between the RFID chip and the rest of the RFID tag, including the microcontroller, the power management circuit, and magnetic field sensor, is presented and discussed in detail. The power management integrated circuit (P2110B) is used to power the microcontroller, the Hall effect sensor, and the RFID chip, as shown in Figure 8. The microcontroller (PIC18F47Q10) converts the analog output of the Hall effect sensor to digital format and writes it to the RFID chip memory through a serial peripheral interface (SPI). Figure 8 shows the communication path of the components of the RFID tag that are powered by the power harvester.

### 4.1. Power Management

The Powercast P2110B is an RF power harvester designed to convert the RF energy emitted by the RFID reader to direct current (dc) power with a maximum achievable efficiency of 80% [31]. The harvested dc power is stored in an external $C_{\mathrm{ext}}=10$ mF capacitor to provide a stable power supply to the other tag components. Once the capacitor is charged to $V_{C\;\max}=1.25$ V, the harvester will boost the voltage to $V_{o}=3.3$ V to supply the power to the microcontroller, the Hall effect sensor, and the RFIC chip. The P2110B power management system includes a low-voltage dropout regulator. If the voltage level of the external capacitor drops below $V_{C\;\min}=1$ V, the circuit will cut off the power supply to the external components. The regulator will resume supplying the power once the voltage level of the external capacitor reaches $V_{C\;\max}=1.25$ V. The total energy stored during the charging period for our design is

(1) $$E_{T}=\frac{1}{2}C_{\mathrm{ext}}\left(V_{C\;\max}^{2}-V_{C\;\min}^{2}\right)=2.8\;\mathrm{mJ}.$$

**Figure 9:**
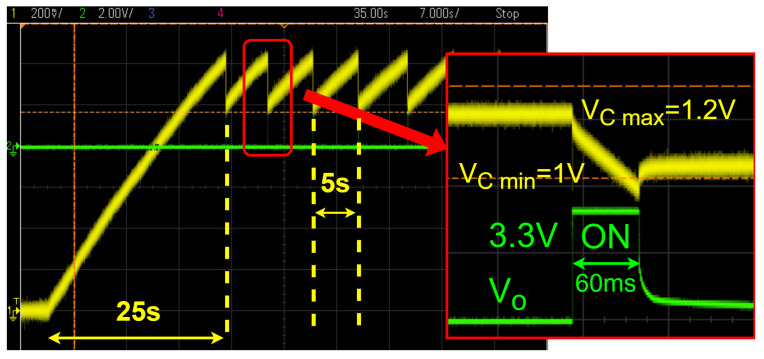
Measured voltage of the external capacitor (yellow trace) during the initial charging time (25 s) and subsequent recharging time (5 s) after sensor operation. The output voltage of the regulator (green trace) is fixed at 3.3 V for 60 ms, during which magnetic field sampling occurs and the external capacitor discharges.

**Figure 10:**
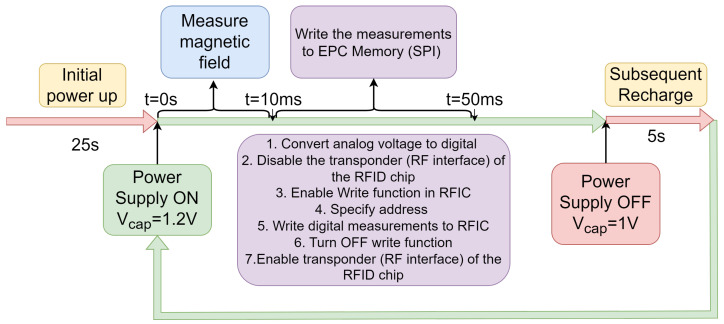
Operation timeline diagram during the 60 ms time window when the output of the Hall effect sensor is read and written into the EPC memory of the RFID chip. During this time, the transponder of the RFID chip is disabled, making it undetectable until the measurement of the magnetic field and transfer of the data to the RFID chip is completed.

This is the maximum energy that can be consumed during the magnetic field measurement process. Here, $E_{T}$ is the the total energy stored during the 5 s recharging time. The maximum operation time (i.e., the time during which the power supply is on) and the power consumed during each measurement are [31]

(2) $$t_{ON}=\frac{C_{\mathrm{ext}}}{15\;V_{o}\;I_{o}}\approx 60\;\mathrm{ms},$$

(3) $$P_{ON}=\frac{E_{T}}{t_{\mathrm{ON}}}\approx 46.8\;\mathrm{mW},$$

where $I_{o}\approx 14.2$ mA is the measured current required by all components energized by the power harvester. The operation time can be adjusted by employing a different capacitor and depends on the current needed to drive the circuit. The process of charging/discharging $C_{\mathrm{ext}}$ is shown in Figure 9. In this case, the distance between the RFID sensor and the RFID reader is 70 cm. The yellow trace shows the voltage of $C_{\mathrm{ext}}$ varying between $V_{C\;\min}=1$ V and $V_{C\;\max}=1.25$ V, and the green trace shows the output voltage $V_{o}$ of the power harvester when it is “ON” at $V_{o}=3.3$ V for 60 ms. For a distance of 70 cm between the sensor and the reader, the initial charging and subsequent recharge times are 25 s and 5 s, respectively. Figure 10 shows the operation timeline after charging is complete. During the 60 ms window of the “ON” time, the microcontroller waits 10 ms for the output of the Hall effect sensor to stabilize. Next, the microcontroller converts the analog voltage to digital and stores the value. Then, the microcontroller will disable the transponder (RF interface) of the RFID sensor, which makes the sensor undetectable by the RFID reader. The digital sensor data are then written to the EPC memory of the RFID chip. After completing the data transfer, the microcontroller enables the transponder, and the RFID sensor becomes detectable again.

### 4.2. Link Budget and Efficiency

The RFID reader used in this work is capable of transmitting a continuous RF signal of 27 dBm power. The RFID reader requires a minimum received power from the tag’s modulated backscattered signal of −66 dbm. Using a free space assumption, the maximum readable distance between the tag and the reader is determined using [32]

(4) $$P_{r}^{R}=P_{t}^{R}e{\left(G^{R}pzG^{T}\right)}^{2}{\left(\frac{\lambda }{4\pi r}\right)}^{4}$$

where $P_{t}^{R}$ is the transmitted power from the RFID reader, $G^{R}$ is the gain of the reader antenna, *p* is the polarization efficiency, *e* is the modulation efficiency, *z* is the matching coefficient, $G^{T}$ is the gain of the RFID tag antenna, λ is the wavelength at 915 MHz, and *r* is the distance between the RFID reader and the tag. The ideal maximum distance between the RFID tag and the RFID reader is r=5.1 m, but, due to mismatch between the tag antenna and the chip and multi-path loss, a maximum readable distance of r≈2 m was measured in the lab. As long as the tag is in the detectable range (i.e., r<2 m), the reading function of the tag is not impacted. However, the charging time and the subsequent recharge times are significantly affected by the distance. For r=70 cm, the initial charge time and the subsequent recharging time are 25 and 5 s, respectively. When the distance increases to r=1 m, the initial charging time increases to 45 s, while the subsequent recharge takes 10 s. At r=2 m, the initial charging time is 180 s, and the subsequent recharge time increases to 45 s.

The received power at the input of the power harvester and the output power of the power harvester can be determined using [32]

(5) $$P_{\mathrm{in}}=P_{t}^{R}G^{R}pezG^{T}{\left(\frac{\lambda }{4\pi r}\right)}^{2}$$

(6) $$P_{\mathrm{out}}=\frac{E_{T}}{t_{\mathrm{recharge}}}.$$

For a distance of r=70 cm, the received input power to the harvester is $P_{\mathrm{in}}=0.71$ mW. Based on the energy stored (see Equation (1)) and the measured recharge time, the power at the output of the harvester is $P_{\mathrm{out}}=0.56$ mW, resulting in efficiency of $P_{\mathrm{out}}/P_{\mathrm{in}}=78\%$. The power required by each component of the RFID sensor during magnetic field sensing is provided in Table 2.

### 4.3. Hall Effect Sensor

**Figure 11:**
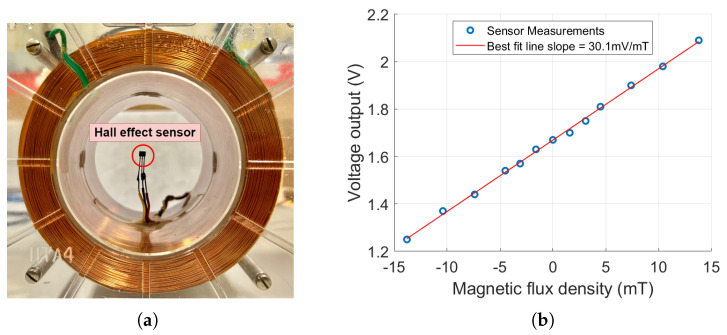
(**a**) Hall effect sensor calibration setup. (**b**) Hall voltage as a function of the magnetic flux density.

A Hall effect sensor is a semiconductor device that detects the presence and strength of a magnetic field [33]. The Hall voltage is linearly proportional to the strength of the magnetic field. The Hall effect sensor DRV 5055-A2 [34], integrated with the developed RFID tag, provides high sensitivity and a wide dynamic linear range. The Hall effect sensor was tested and calibrated using a 3400-turn solenoid with a dc current source, as shown in Figure 11a. The measured sensor output voltage as a function of the magnetic flux density for a supply voltage of 3.3 V is shown in Figure 11b. The measured sensitivity is 30.1 mV/mT over a dynamic range of −14 mT to +14 mT. As shown in Figure 11b, knowing the current of the coil, the magnetic flux density values of the horizontal axis were determined using COMSOL Multiphysics 6.0, a commercial finite element software program by COMSOL Inc. (Burlington, MA, USA).

## 5. Experimental Setup for Testing of the RFID Magnetic Field Sensor

**Figure 12:**
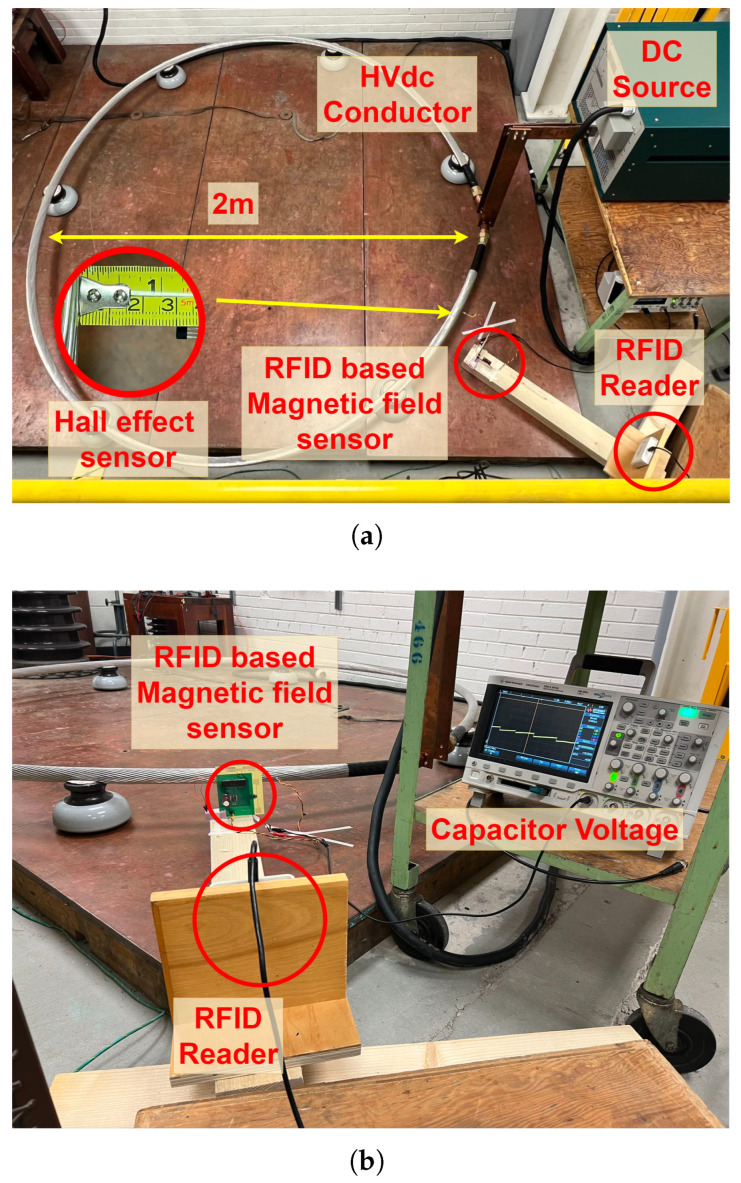
Experimental setup for the measurement of the magnetic flux density generated by a dc current flowing through a HVdc conductor: (**a**) top view and (**b**) side view.

The fabricated sensor was tested in the McMath High Voltage Lab at the University of Manitoba. A test setup simulating the same conditions as a HVdc transmission line was developed to evaluate the sensor’s performance (see Figure 12). A dc source capable of generating a short-circuit current of up to 1200 A was connected to a conductor, similar to those that are employed in the 500 kV HVdc transmission lines in Manitoba. The HVdc conductor had a diameter of 4 cm, and the loop created by the conductor had a diameter of 2 m. The sensing system was placed near the conductor. The distance between the Hall effect sensor and the outer conductor surface was 3 cm. The RFID reader was placed 1 m away from the RFID sensor. The distance between the RFID-based sensor and the RFID reader does not affect the accuracy of magnetic field measurements, but it does influence the charging time of the sensor.

## 6. Results and Discussion

**Figure 13:**
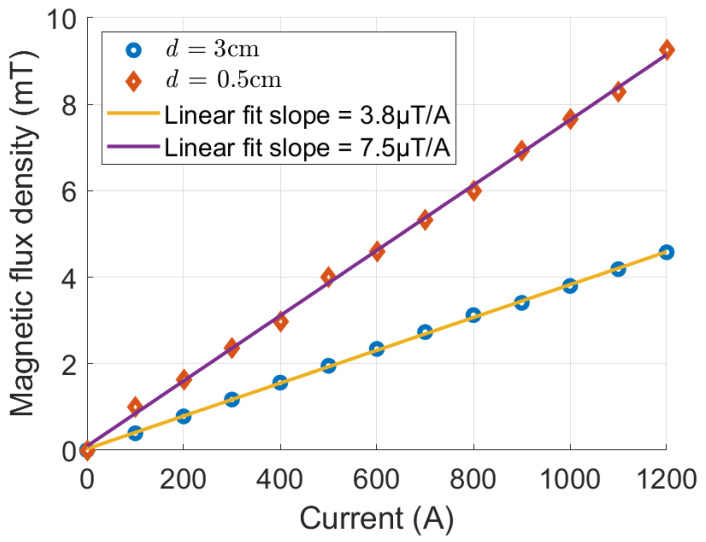
Magnetic flux density measured using RFID sensor for conductor surface-to-sensor distance of d=0.5 cm and d=3 cm. Sensitivity of 3.8μT/A and 7.5μT/A is shown, respectively.

Experiments were performed for a 1 m reader–tag distance and for different sensor–conductor distances. The current flowing through the HVdc conductor was varied from 0 to 1200 A. The measured magnetic flux densities are shown in Figure 13 for a conductor surface-to-sensor distance of d=0.5 and 3 cm. The measured magnetic flux densities show that the sensor has a highly linear relationship between the magnetic flux density and the current. The maximum deviation from a linear fit is 0.15 mT and 0.05 mT, respectively. The sensitivity of the RFID sensor is 7.5 μT/A for d=0.5 cm and 3.8 μT/A for d=3 cm. As shown in Figure 13, when the sensor-to-conductor surface distance reduces from 3 cm to 0.5 cm (equivalent to a conductor center-to-sensor distance of 5 cm to 2.5 cm), the measured magnetic flux density increases by a factor of 2, following the expected 1/r behavior of the magnetic flux density near an infinitely long conductor. The differences between the linear fit and the magnetic flux density measurements are possibly due to inaccuracies in the Hall effect sensor’s position and orientation. Furthermore, the microcontroller’s ADC requires an accurate reference voltage of 3.3 V supplied by the power harvester’s output voltage, which can be noisy. It should be noted that, for a distance of 1 m between the RFID sensor and the RFID reader, the initial charging time and the subsequent recharge time are around 45 s and 10 s, respectively (see Figure 9).

The accuracy of the RFID sensor is affected by the Hall effect sensor error, errors in its position and orientation with respect to the transmission line, and the quantization error of the ADC. The error due to the Hall effect sensor is 1% or ≈0.3 mV [34]. For the 10-bit ADC (1 bit for the sign) and a reference voltage of 3.3 V, the quantization error is 6.4 mV, which dominates the error of the Hall effect sensor. This translates to an inaccuracy of ±0.2 mT or ±30 A. The position/orientation error will result in a linear scaling error that can be accounted for by calibration.

**Figure 14:**
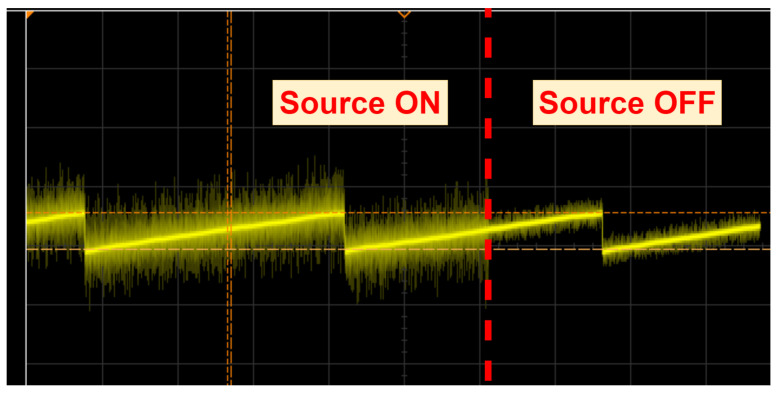
Measured sensor capacitor voltage showing the noise when the dc source is ON ($V_{\mathrm{noise},\mathrm{pp}}\approx 0.75$ V) and OFF ($V_{\mathrm{noise},\mathrm{pp}}\approx 0.2$ V).

There is a high level of electromagnetic noise expected near high-voltage equipment such as the switching circuit in a power station. In our test scenario, we performed measurements next to the dc source used to generate the dc current. Figure 14 shows the added noise measured across the supercapacitor when the dc source is on. This demonstrates the ability of the sensor to operate in an environment with a high level of electromagnetic interference.

## 7. Conclusions

A UHF RFID-based sensor was designed, fabricated, and tested for the measurement of the dc magnetic flux density from HVdc equipment. The RFID-based sensor includes an integrated power harvester that collects the energy required for the operation of the RFID tag from the emitted energy by the RFID reader. Two separate antennas were designed: one for the communication between the RFID chip and the RFID reader and one for power harvesting. A microcontroller was employed to manage data communication between the Hall effect sensor and the RFID chip. The entire batteryless RFID tag was fabricated on a single PCB, which enables a compact design. The fabricated RFID-based magnetic field sensor was tested in a setup that resembled an HVdc transmission line and carrying a maximum dc current of 1200 A. The sensor’s performance was evaluated at different sensor–conductor separations for a 1 m reader-to-sensor distance. Sensitivities of 7.5 μT/A and 3.8 μT/A were measured for sensor–conductor separations of 0.5 and 3 cm, respectively. The sensor has a dynamic range of 0 to 9 mT, corresponding to a dc current of 0 to 1200 A. The sensor demonstrates a maximum deviation from a linear fit of 0.15 mT and 0.05 mT for a 0.5 and 3 cm tag-to-conductor distance. There is a trade-off between the interrogation distance and the charge/recharge time. For a distance of 1 m between the RFID reader and the RFID-based sensor, an initial charging and a subsequent recharge time of 45 s and 10 s were measured. The performance of our sensor was evaluated in a controlled lab environment. The source of noise during evaluation was primarily due to a single high current supply. The noise could be much stronger in the field, such as in a converter station. Our RFID-based magnetic field sensor does not compensate for temperature affects caused by the heating of transmission line conductors under high current conditions. Additionally, the specifications of the Hall effect device are tailored to the desired dynamic range.

In the RFID sensor described in this paper, the power is obtained from the RFID reader. The RFID reader’s effective isotropic radiated power (EIRP) is limited to 36 dBm, which restricts the available power to operate the tag’s components and/or the interrogation distance. Using an RFID tag antenna with higher directivity could enable a larger interrogation distance. Incorporating multiple power harvesting schemes that can draw power from diverse ambient sources could enhance the system’s overall functionality. Future developments should be assessed by evaluating the reader–tag data communication reliability and power harvesting efficiency for different reader–tag distances, the immunity of the system to different levels of noise, and the effects of obstacles that may cause multipath interference.

## Figures and Tables

**Table 1:** Wireless sensors for overhead transmission line monitoring.

Parameter	Type of Line	Type of Sensor	Power Source	Ref.
Magnetic field	AC and DC	MEMS	Solar energy	[23]
Current and sag	AC	Hall effect sensor	Rogowski coil	[24]
Current	AC	Multiple sensor	Electromechanical harvester	[25]
Vibration	AC and DC	Accelerometer	Solar energy	[26]
Vibration	AC and DC	Accelerometer	Battery	[27]
Magnetic field	DC	Hall effect sensor	RFID reader	This work

**Table 2:** Power required by each component of the sensor during magnetic field sensing.

Component	Supply Voltage	Required Power
Microcontroller	3.3 V	19.2 mW
Hall effect sensor	3.3 V	19.8 mW
RFID chip	3.3 V	3.3 mW

**Figure A1:**
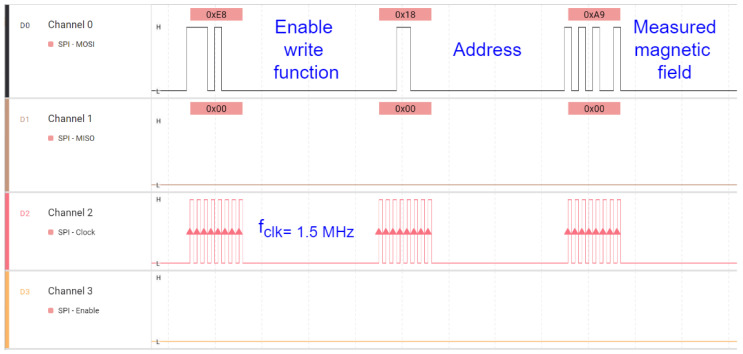
Logic signal analyzer scope.

## Data Availability

Data are contained within the article.

## Appendix A. Design of SPI Interface

The microcontroller is the most important part in the digital function block of the sensor tag design. In this design, a Microchip ultra-low-power 10-bit microcontroller, the PIC18F47Q10, is used. The maximum current drawn is 4.4 mA with a 3.3 V power supply. The PIC18F47Q10 microcontroller also includes an array of additional features that are suitable for the implementation of the RFID-based magnetic field sensor. It operates at speeds of up to 64 MHz and includes a range of peripherals, such as multiple serial communication interfaces (USART, SPI, I2C). The clock speed can be modified by setting a specific register in the chip. It also includes multiple timers and a 10-bit analog-to-digital converter (ADC).This combination of features and flexibility makes the PIC18F47Q10 suitable for applications implementing the RFID-based magnetic field sensor.

To encode the measured magnetic field information, the ADC will be used to convert the voltage output from the Hall effect sensor. The ADC is configured to sample the analog input voltage at a 3.3 V reference voltage. This means that the ADC only sample voltages between 0V and 3.3 V, transferring them to corresponding 10-bit binary values within this range. During each sampling period, the ADC compares the input voltage to the internal reference voltage to determine its approximate digital value. The resulting output is a 10-bit digital code, which provides a resolution of 1024 distinct values from 0 to 3.3 V. This ensures that any small changes in the analog signal can be accurately captured. This digital code is then transmitted to the RFID chip.

To write the digital code to the EPC memory of the RFID chip, it is first converted to a hexadecimal format for compatibility with the RFID memory structure. Then, the hexadecimal number is transmitted to the RFID chip over the SPI bus, with the RFID chip configured as an SPI slave device, allowing it to receive data directly from the controlling microcontroller. The EM4325 RFID chip receives these data and stores them in a designated memory location controlled by the microcontroller, ensuring that the measured magnetic field data are correctly logged in the EPC memory.

In SPI slave mode, this device is configured to accept an SPI clock that operates independently of its internal processes. The SPI polarity and phase are configured through the SPI control word. When the SPI master clock (SCLK) operates, the maximum frequency is 2 MHz for battery-assisted passive mode. The SPI master needs to release the chip select (CS) line for at least 15 µs between commands. The device’s maximum response time to an SPI command is 20 ms, and any response will start with a data value of ‘1’. The following example shown in Figure A1 demonstrates how this device operates as an SPI slave to communicate with an external SPI master [29].

To verify that the digital data emerging from the microcontroller are correct, a logic analyzer is used to visualize the digital signal. As shown in Figure A1, the clock used for the SPI interface, which should not be larger than 2 MHz, is set to be 1.5 MHz [29]. The master output slave input (MOSI) bus is used to transmit three bytes: the command to enable writing functions, the address where the data will be stored, and the measured magnetic field data.
